# Polyamide Backbone Modified Cell Targeting and Penetrating Peptides in Cancer Detection and Treatment

**DOI:** 10.3389/fchem.2020.00218

**Published:** 2020-03-31

**Authors:** Sunil S. Shah, Nelson Casanova, Gina Antuono, David Sabatino

**Affiliations:** Department of Chemistry and Biochemistry, Seton Hall University, South Orange, NJ, United States

**Keywords:** cell penetrating peptides, cancer targeting peptides, polyamide backbone modified peptides, peptidomimetics, cancer detection, cancer treatment, cancer theranostics

## Abstract

Cell penetrating and targeting peptides (CPPs and CTPs) encompass an important class of biochemically active peptides owning the capabilities of targeting and translocating within selected cell types. As such, they have been widely used in the delivery of imaging and therapeutic agents for the diagnosis and treatment of various diseases, especially in cancer. Despite their potential utility, first generation CTPs and CPPs based on the native peptide sequences are limited by poor biological and pharmacological properties, thereby restricting their efficacy. Therefore, medicinal chemistry approaches have been designed and developed to construct related peptidomimetics. Of specific interest herein, are the design applications which modify the polyamide backbone of lead CTPs and CPPs. These modifications aim to improve the biochemical characteristics of the native peptide sequence in order to enhance its diagnostic and therapeutic capabilities. This review will focus on a selected set of cell penetrating and targeting peptides and their related peptidomimetics whose polyamide backbone has been modified in order to improve their applications in cancer detection and treatment.

## Introduction

Cell targeting and penetrating peptides (CTPs and CPPs) are a class of peptides that have the capabilities of targeting and translocating within specific cell types. As such, they are particularly well-suited for the targeted delivery of imaging and/or therapeutic agents, because of their specific accumulation in targeted cell lines, tissues or even disease-bearing *in vivo* models (Tripathi et al., 2018). This targeted delivery effect amplifies the detection capabilities of the biological probe or the therapeutic index of the drug for more effective and precise diagnosis and therapy at the localized site of malignancy (Borrelli et al., 2018). More specifically, CPPs and CTPs are about 4–30 amino acids in length, derived from biologically active motifs found in proteins or from selection techniques that have ushered in unique peptides with the abilities to target cell surface biological markers (e.g., cell surface receptors) and translocate within cells by a variety of cell uptake mechanisms (Habault and Poyet, 2019). As such, CTPs and CPPs have been applied to transport various cargos such as nucleic acids (McClorey and Banerjee, 2018), proteins (Kristensen et al., 2016), imaging probes (Juliano et al., 2009), and drugs (Feni and Neundorf, 2017) into selected cell lines for activity.

In cancer, tumor cells have cell surface receptors that are up-regulated and overexpressed within the plasma membrane where they signal tumor initiation, progression, spread and resistance toward treatment. These biological markers are absent or present to a lower extent in normal cells and can be exploited to deliver various cargos specifically to tumor cells for cancer-targeting detection and treatment strategies (Goossens et al., 2015). Thus, cancer-targeting peptides are a class of CTPs that have been successfully used to transport various anti-cancer drugs, imaging agents, and toxins for specific imaging and treatment at the localized tumor site (Liu et al., 2017). Despite their utility, CTPs and CPPs are limited by poor pharmacological properties which restrict their translation into the clinic (Reissmann, 2014).

## Polyamide Backbone Modifications in Peptides and Peptidomimetics

**Figure 1:**
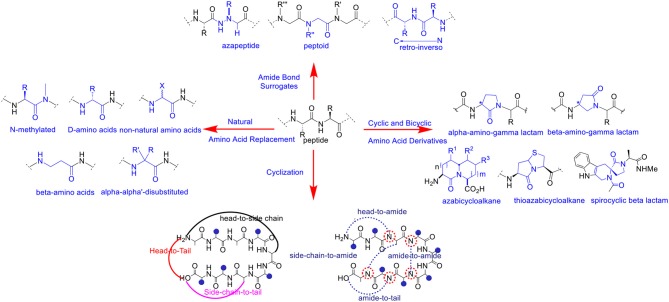
Selected examples of polyamide backbone modifications in peptides and peptidomimetics.

A variety of chemical modifications have been designed and developed as peptide bond surrogates (Figure 1) in order to enhance and optimize the biological effects of *de novo* peptide analogs. Interestingly, substitution or replacement of the polyamide backbone has resulted in profound effects on hybridization, chirality, and conformation of the peptide bond. The native peptide bond resonates with significant free energy barriers (65–76 kJ/mol) for bond rotation and trans-cis interconversions dependent on the chemical nature of the amino acid residues (Scherer et al., 1998). The Cα chirality projecting in either naturally occurring L or unusual D orientations affects the 3D projection of the side chain and the dihedral angles (φ/ψ) of the polyamide backbone. The latter is a critical requirement in pre-organizing stable peptide secondary structures and folding into higher-order assemblies (Garcia et al., 2018). Thus, chemical modification of the polyamide backbone can trigger profound changes in molecular chirality, hybridization, conformation and in the self-assembly of peptide structures and nanoparticles.

Peptide bond surrogates include but are not limited to azapeptides which replace the Cα for a nitrogen atom resulting in a semicarbazide which profoundly effects molecular chirality, hybridization, and conformation of the pseudo peptide bond (Proulx et al., 2011; Danelius et al., 2019). As such, azapeptides have been shown to stabilize β-turn conformations in bio-active peptides used for biomedical applications (Chingle et al., 2017). This includes azapeptide ligands that can modulate unregulated receptor tyrosine kinase signaling in cancer (Kurian et al., 2014). Peptoids are a class of biomimetic peptides in which the peptide backbone is replaced with repeating *N*-substituted glycine units (Proulx et al., 2015). Although peptoids are devoid of chirality, the introduction of chiral *N*-substituted side chain groups provides significant effects in adapting local molecular geometries and higher order structural self-assemblies for a wide range of biomedical and nanomaterials applications (Tran et al., 2011; Laursen et al., 2013). Of specific interest herein are the representative peptoid case studies based on the diagnosis and treatment of cancer (Bhowmik et al., 2017). Retro-inverso peptides consisting of D-amino acid residues in reversed sequence adopt side chain topologies similar to the parent peptide but with inverted amide bonds. These modifications have served to improve peptide metabolic stability but with limited capabilities of adopting bio-active peptide α-helical structures necessary for protein binding interactions and downstream biological signaling such as silencing gene expression in certain cancer types (Li et al., 2010, 2015). Main chain replacement with peptide amide bond isosteres (Williams et al., 1994; Hart and Etzkorn, 1999; Chen et al., 2017) have also resulted in important contributions in varying peptide conformation and stereoelectronic effects which impacts peptide bioactivity against receptor targets in cancer (Crombez et al., 2009). Head-to-tail cyclizations have also resulted in the ligation of the polyamide backbone N-to-C-termini resulting in macrocyclic peptides with improved metabolic stability, cell permeability and receptor targeting capabilities (Janssen et al., 2002; Marinelli et al., 2003; Ye et al., 2006; Smith et al., 2011, 2013; White and Yudin, 2011; Koopmanschap et al., 2014; Shirazi et al., 2014a,b; Vinogradov et al., 2019). Taken together, these selected peptide bond modifications have served to improve the physicochemical and structural properties of the native peptide sequence in order to generate more potent peptidomimetics for anti-cancer applications.

Similarly, amino acid substitution by an un-natural amino acid residue has also resulted in polyamide backbone modifications that have improved peptide chemical and biological properties. Replacement of naturally occurring L- for the unusual D-amino acids are among the most common substitutions. This change in chirality at the Cα position has resulted in peptide analogs with improved resistance to proteolysis while retaining the biological activity of the parent peptide (Tünnemann et al., 2008; Hanagata, 2012; Chung et al., 2017; Manabe and Kawasaki, 2017). Modified linear aliphatic amino acids such β, γ and α,α-disubstituted amino acids have provided stabilizing effects on peptide helical structures resulting in well-defined peptide foldamers with desirable binding properties with receptor targets found in cancer (Horne and Gellman, 2008; Crombez et al., 2009). Comparably, N-alkyl amino acids effectively perturb the H-bonding capabilities of the amido NH group in peptide bonds which in turn effects secondary structure formation and stability (Di Gioia et al., 2016). These structural implications play important roles in modulating ligand-receptor binding interactions, that contribute to high affinity and selective peptide binding to cancer signaling receptors (Marinelli et al., 2003). Cyclic and bicyclic amino acid derivatives (Cluzeau and Lubell, 2004; Proulx and Lubell, 2012; Li et al., 2013; Vezenkov et al., 2017), also including lactam (Jamieson et al., 2009; Vezenkov et al., 2017) and spirocyclic lactam (Lesma et al., 2013) type peptide mimics, in addition to (thio)azabicyclo (Gu et al., 2004)-peptidomimetics have each served as privileged structures most often stabilizing β-turn conformations that result in high affinity and discriminate binding to cancer receptors (Auzzas et al., 2010). Interestingly, lactam-based polymer foldamers enabled the delivery of various cargo (e.g., detection probes and drugs) across cell membranes of cancer cells while exhibiting proteolytic stability (Vezenkov et al., 2017). Taken altogether, peptide backbone modifications have served to improve the biophysical and biological properties of peptidomimetics. The translation of modified peptide ligands into more potent anti-cancer agents are among the most widespread applications. The selected examples highlighted herein will serve to represent the importance of polyamide backbone modifications in cell targeting and penetrating peptides that have been exploited for the detection and treatment of cancer.

## Rationale for Backbone Modifications in Cell Penetrating/Targeting Peptides

**Figure 2:**
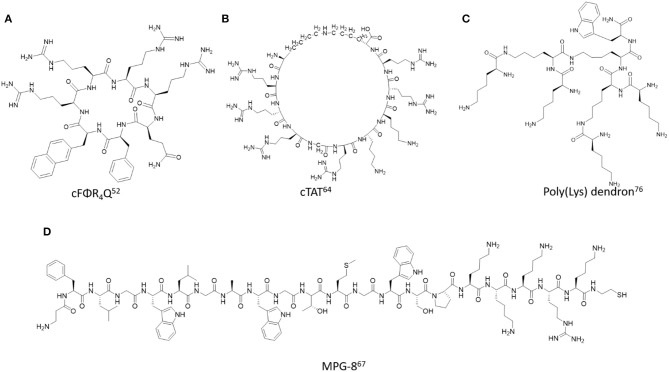
Representative examples of backbone modified cell penetrating peptides described in this study. **(A)** cFΦR_4_Q (Mandal et al., 2011), **(B)** cTAT (Nischan et al., 2015), **(C)** Poly(Lys) dendron (Janiszewska et al., 2016), **(D)** MPG-8 (Crombez et al., 2009).

**Figure 3:**
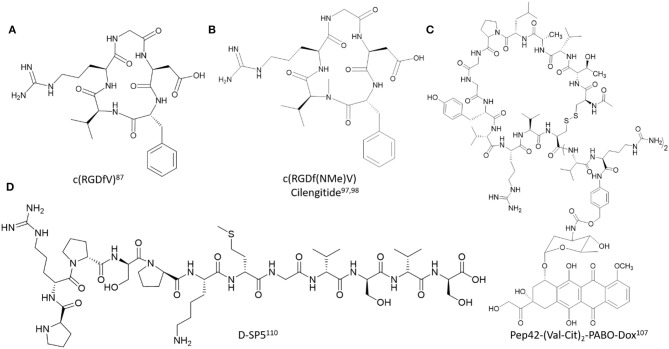
Representative examples of polyamide backbone modified cell-targeting peptides described in this study. **(A)** c(RGDfV) (Kessler, 1997), **(B)** c(RGDf(NMe)V) Cilengitide (Dechantsreiter et al., 1999; Marinelli et al., 2003), **(C)** Pep42-(Val-Cit)_2_-PABO-Dox (Yoneda et al., 2008), **(D)** D-SP5 (Li et al., 2015).

In order to improve the biological properties of cell penetrating and targeting peptides (CPPs and CTPs) several biochemical strategies have been developed to design and construct related peptidomimetics. Of specific interest herein, are the design applications which modify the polyamide backbone composition of lead cell penetrating and targeting peptides (Figures 2, 3). These modifications aim to improve the physicochemical characteristics of the native peptide sequence while retaining its beneficial biological and potentially therapeutic properties. More specifically, these structural modifications may ameliorate peptide solubility and bioavailability in biological media while also improving its serum stability toward endogenous peptidases and proteases (Gentilucci et al., 2010). The latter has shown to effectively improve the resident time of the peptide at the localized biological site for extended duration of action, thereby increasing its therapeutic index (Chen et al., 2015). Moreover, reduction of immunostimulatory effects and off-target toxicity associated with non-selective and low affinity binding to receptor targets has facilitated the translation of lead sequences into effective peptide-based anti-cancer drugs (Luther et al., 2017). Medicinal chemistry has been implemented in the design and development of peptidomimetics in which the pharmacophoric region responsible for bioactivity has been modified to improve structure-activity relationships (Blaskovich, 2016). In this section, we will focus on a selected set of examples which highlights the chemistry, characterization and application of peptidomimetics whose polyamide backbone has been altered in order to improve the biological responses of CPPs and CTPs in cancer detection and treatment (Table 1).

**Table 1:** Selected examples of backbone modified CPPs and CTPs described in this review.

**Peptide**	**Sequence**	**Modification**	**Application**	**References**
CPPs in cancer detection and treatment				
1. Poly(arginine)	*r_n_* = 3–12	D-Arg	Cell uptake studies and siRNA delivery	Tünnemann et al., 2008; Chung et al., 2017
2. cFΦR_4_Q	Cyclo(FΦRRRRQ)^a^	Cyclic-poly(Arg)	FITC delivery	Qian et al., 2013
3. [WR]_5_	Cyclo(WWWWWRRRRR)	Cyclic-poly(Arg)	Au and Se nanoparticles for drug delivery	Shirazi et al., 2014a,b
4. cTAT	Cyclo(GRKKRRQRRRPQ)	Cyclic-poly(Arg)	GFP delivery	Nischan et al., 2015
5. MPG-8	bAFLGWLGAWGTMGWSPKKKRK-Cya	β-Ala^b^, Cya^c^	siRNA delivery	Crombez et al., 2009
6. Poly(lysine)	${\text{k}}_{\text{n}}^{\text{d}}$	D-Lys	CpG^g^ delivery	Hanagata, 2012
7. Poly(lysine)	(Gly–Lys_63_(NH_2_)_64_)	Poly(lysine) dendron	pDNA and siRNA delivery	Al-Jamal et al., 2006; Janiszewska et al., 2016
CTPs in cancer detection and treatment				
8. RGD	Cyclo(RGDfV)	Cyclic-RGD D-Phe	Radiotracer and near IR label delivery	Janssen et al., 2002; Ye et al., 2006
9. RGD	Cyclo(RGDf[N-Me]V)	Cyclic-RGD D-Phe, N-Me Val	α_v_β_3_ integrin receptor inhibitor	Marinelli et al., 2003
10. Pep-42	CTVALPGGYVRVC-Val-Cit-PABA	Cit^e^, PABA^f^	Drug delivery	Yoneda et al., 2008
11. D-SP5	prpspkmgvsvs	Retro-inverso D-peptide	pDNA delivery	Li et al., 2015

a *Where Φ is L-2-naphthylalanine*.

b *β-Ala is β-alanine*.

c *Cya is cysteamide*.

d *Variable number of D-Lys units*.

e *Cit is citrulline*.

f *PABA is p-aminobenzyl alcohol*.

g *CpG is cytosine-phosphate-guanosine*.

## Selected Examples of Polyamide Backbone Modified Cell Penetrating Peptides

### Poly(arginine)

The poly(arginine) peptides encompass a special class of cell penetrating peptides (CPPs) whose structure-function relationships have facilitated a wide range of cell biology applications. Their polar, hydrophilic and polycationic character allows them to interact favorably with cell membranes, resulting in efficient endocytic cellular translocation, trafficking, and endosomal escape for biological activity (Mitchell et al., 2000; Zorko and Langel, 2005). Interestingly, their cell uptake capabilities are dependent on the length and structure of the poly(arginine) sequence, with optimal cellular delivery often associated with 6-12 arginines (Alhakamy and Berkland, 2013). Their favorable uptake properties in a wide range of cell types and also within *in vivo* models has enabled poly(arginine) sequences to be functionalized or condensed with varying cargos. These include biologics such as nucleic acids (Kim et al., 2010) and proteins (Futaki et al., 2001), detection probes (Guo et al., 2007) and therapeutic agents (Borrelli et al., 2018) that have ushered in a wide range of applications in molecular cell biology and medicinal chemistry. Despite their potential, native poly(arginine) sequences are limited by poor metabolic stability, non-specific cell binding and uptake which has restricted their optimal biological activities *in vitro* and *in vivo* (Vives, 2005).

Substitution of naturally occurring L- for D-arginine (Arg) residues are among the most prevalent modifications of poly(arginine) peptides. This simple modification is most often incorporated by Fmoc-based solid phase peptide synthesis (Fmoc-SPPS) using commercially available Fmoc-D-Arg(Pbf)-OH building blocks to construct the poly(arginine) sequence on solid support. The judicious selection of the solid support in this particular case is based on the more polar poly(ethylene)glycol (PEG) resin, that has shown increased capabilities of swelling favorably in organic solvent (DMF) and reagents (TFA) typically used in the Fmoc-SPPS method (García-Martín et al., 2006; Joseph et al., 2014). Efficient coupling with 2-(6-Chloro-1-H-benzotriazole-1-yl)-1,1,3,3-tetramethylaminium hexafluorophosphate (HCTU) as activator and N-methylmorpholine (NMM) as base has resulted in successful synthesis of poly(arginine) peptides of varying lengths and stereochemistry at the Cα position. Furthermore, poly(D-arginine) peptides have flexible peptide structures in solution, dependent on the length of the poly(arginine) sequence, sample concentration, temperature, in addition to the type of solvent or buffer and its corresponding ionic strength and pH for solubilizing the peptide in solution (Joseph et al., 2014). Circular dichroism (CD) spectroscopy has been most frequently used to detect the type of secondary structure and its stability in solution. Poly(D-arginine) peptides may also adopt helical and turn-type secondary structures, that are contingent on sample concentration, temperature, pH, solvent, or buffer conditions. Consequently, D-Arg peptides have shown excellent cell permeability properties and extended half-lives in serum, living cells and in animal models. Their translational capabilities, from cell-based *to in vivo* applications, are associated with their biocompatibility and extended duration of action making them ideal candidates for investigating delivery applications associated with the mechanisms of cell uptake, intracellular trafficking and localization (Tünnemann et al., 2008). Poly(D-arginine) peptides have also been functionalized with PEG and used for the transport of detection bioprobes in cells and *in vivo* (Chung et al., 2017). The latter makes them especially useful in the diagnosis of malignant cell types, such as cancer. Furthermore, D-Arg peptides have also been used for the transport of anti-cancer agents such as short interfering RNA (siRNA) that can silence oncogene mRNA expression and trigger cell death in cancer cell models and within tumor bearing mice (Won et al., 2011).

Cyclic-Arg peptides are another common example of modified poly(arginine) peptides. More specifically, cyclization methods for constructing head-to-tail cyclic poly(arginine) sequences have been developed to improve the biological properties of the native sequence. For example, the cyclo(FΦRRRRQ) (cFΦR_4_Q, where Φ is L-2-naphthylalanine) was synthesized by Fmoc SPPS using benzotriazol-1-yl-oxytripyrrolidinophosphonium hexafluorophosphate/hydroxybenzotriazole/*N, N*-diisopropylethylamine (PyBOP/HOBt/DIPEA) as coupling conditions to effect the ***N***to ***C***-terminal cyclization (Qian et al., 2014). The cyclic peptides displayed serum stability, delivery efficiency, and minimal toxicity. The cyclic Arg-rich peptides were also conjugated with fluorescent reporters such as FITC to investigate the mechanism associated with cell internalization, cargo delivery, and endosomal escape for downstream biological assays such as the detection of intracellular protein tyrosine phosphatase activities (Qian et al., 2013). In a related application, the cyclic Arg-rich peptide containing alternating arginine and tryptophan residues, namely [WR]_5_ when fluorescently labeled was found to improve intracellular delivery and nuclear localization in colon adenocarcinoma, breast carcinoma, and human ovarian adenocarcinoma with respect to the parent linear sequence (Mandal et al., 2011). Peptide head-to-tail cyclization was optimized with the mild carbodiimide-based coupling conditions, *N, N*′*-*diisopropylcarbodiimide/1-hydroxy-7-azabenzotriazole/*N, N*′-dimethylformamide/dichloromethane, (DIC/HOAT/DMF/DCM), which produced predominantly the cyclic [WR]_5_ peptide which was purified by RP HPLC and confirmed by MALDI TOF mass spectrometry (Shirazi et al., 2013). Moreover, formulation of [WR]_5_ with gold (Au) and selenium (Se) produced discrete nanoparticles in the size range of 100–300 nm according to transmission electron microscopy (TEM), scanning electron microscopy (SEM) and dynamic light scattering (DLS) (Shirazi et al., 2014a,b). The nanoparticles were functionalized with fluorescently-labeled phosphopeptides to track cell uptake and were loaded with a wide range of anti-cancer drugs, such as siRNA, doxorubicin, gemcitabine, clofarabine, etoposide, camptothecin, irinotecan, epirubicin, fludarabine, dasatinib, and paclitaxel which each exhibited more potent toxicity in SK-OV-3 cancer cells when used in combination.

### TAT

The TAT peptide is a short CPP derived from the transactivator of transcription (TAT) fusion transduction domain of the HIV retrovirus (Del Gaizo Moore and Payne, 2004). The TAT peptide (GRKKRRQRRRPQ) is an Arg-rich, polycationic, 12-amino acid containing peptide that has been labeled with a green fluorescent protein (GFP) bioprobe in order to evaluate its mechanism of cellular translocation. Interestingly, the CPP was found to interact with negatively charged membrane phospholipids causing a flip in phosphatidylserine (PS) from the inner to outer cell membrane during transduction. The exposure of PS on the outer plasma membrane has long been considered a unique feature of apoptotic cells, however, this mechanism was not found to be apoptosis dependent, but rather related to a novel mechanism for peptide transduction at the cell membrane. Additional mechanistic studies with the Alexa Fluor-488 labeled TAT peptide revealed punctate cytoplasmic distribution of the peptide with similar localization as the endocytic markers transferrin or FM 4-64 (Richard et al., 2003). Furthermore, mild cellular fixation with formaldehyde resulted in redistribution of the TAT peptide to the nucleus, without any significant changes in the co-localization with endocytic markers. Taken together, these results strongly support the involvement of endocytosis as the major route for TAT peptide cell uptake. However, increasing studies have shown that the incorporation of relatively large, charged fluorophores, bulky hydrophobic drugs and large, complex biologics such as siRNA, plasmid DNA, and proteins can lead to significant changes in the cell uptake mechanism of the CPP (Macchi et al., 2015; Akhunzada et al., 2017). Furthermore, in certain cases cell uptake was abrogated when a CPP targeting the prostate membrane antigen receptor (PSMA) was combined with siRNA due to the formation of large, negatively charged particles according to DLS and TEM (Cultrara et al., 2019). Therefore, extensive structural studies are necessary to fully understand the translocation mechanism of the cargo-bound CPP. Structural studies using fluorescence and circular dichroism (CD) spectroscopy was used to study the conformation of the TAT peptide and related analogs in the presence of phospholipids that self-assembled into small unilamellar vesicles (SUVs) of varying charge densities (Ruzza et al., 2010). The results revealed a strong dependence of the peptide-lipid interactions on the nature and composition of vesicles and peptides. The CD spectrum of the TAT peptide in Tris buffer (pH: 6.8) at low temperature (10°C) displayed a strong negative band at around 196 nm and a weak positive band centered around 223 nm. With an increase in temperature (10–50°C), the 223 nm positive band disappeared, while the intensity of negative band at around 196 nm decreased. In addition, an isodichroic point at around 210–215 nm was observed, suggesting that the TAT peptide adopts a combination of different structures, with a low-temperature conformation most closely resembling a type II polyproline (PP_II_) helix. The helical structures and the polycationic nature of CPPs such as the TAT peptide have been found to be important contributing factors for cell uptake and cargo delivery. Therefore, the TAT peptide has been applied in the cell uptake of small nanoparticles, drugs, and biologics for applications in cancer cell-based detection and treatment *in vitro* and *in vivo* (Morshed et al., 2016). In spite of its utility, the widespread biomedical applications of the TAT peptide are still limited by its poor metabolic stability, specificity and short duration of action due to rapid clearance from the target site (Vives, 2005).

Main chain cyclization of the TAT peptide has served to improve its translocation capabilities, especially when associated with the delivery of larger, ionically charged biomacromolecules (Thompson et al., 2012). It is proposed that cyclic TAT (cTAT) displays the Arg side chain guanidinium groups in an extended, distant orientation from the peptide backbone, which in turn, increases surface membrane lipid ionic contacts resulting in enhanced cellular translocation (Lättig-Tünnemann et al., 2011). In a representative example, a backbone modified TAT peptide derivative (rRrGrKkRr) with alternating L-/D- amino acid residues was flanked with orthogonally protected Lys(Alloc) and Glu(Allyl). Following selective side chain deprotection, the reactive Lys amino and Glu carboxylate groups were condensed to the cTAT peptide using HATU/DIPEA as coupling conditions (Nischan et al., 2015). The cTAT peptide was then modified at the *N*-terminus with an amino-functionalized PEG linker which was used to condense azidobutanoic acid to afford the *N*-terminal azide-linked peptide which was finally isolated by RP HPLC and characterized by HRMS. The azido-functionalized cTAT peptide was then conjugated with a recombinant homopropargylglycine green fluorescent protein (GFP) mutant by the copper-catalyzed azide-alkyne cycloaddition to yield the GFP-cTAT bioconjugate. The sample was isolated by RP HPLC, characterized by HRMS, UV Vis and fluorescence spectroscopy. Cell uptake studies in human cervical cancer HeLa cell lines revealed a faster rate and larger accumulation of cTAT-GFP in the cytosol and nucleus compared to the linear TAT-GFP counterpart. In this case, the cell uptake mechanism was evaluated with a macropinocytosis inhibitor at 4°C, which proved that the mode of cell uptake was non-endocytic and energy independent. Taken together, these studies highlight the potential of cTAT to enhance cell uptake efficiency and kinetics for the delivery of large biological cargos, such as proteins.

### MPG

The MPG peptide is a rationally designed 27 amino acid residue containing peptide vector (Ac- GALFLGFLGAAGSTMGAWSQPKKKRKV-Cya) with a cysteamide (Cya) group at the *C*-terminus and an acetyl (Ac) group at the *N*-terminus to improve its cell permeability and stability (Morris et al., 1997). The MPG peptide contains three distinct domains: an *N*-terminal hydrophobic region (GALFLGFLGAAGSTMGA) derived from the fusion sequence of the HIV-1 gp41 (glycoprotein 41), required for cell uptake, a hydrophilic, polycationic domain (KKKRKV) derived from the nuclear localization sequence (NLS) of the simian virus 40 (SV40) large T-antigen which facilitates intracellular localization of negatively charged cargo, such as DNA and RNA, and an internal linker sequence (WSQP), designed to improve flexibility and integrity of the flanking domains. The MPG peptide is stable to degradation, soluble in aqueous media, and buffers and exhibits a flexible structure. The peptide is a random coil in water but adopts a folded α-helix structure with characteristic minimum bands at 210 and 230 nm according to the CD spectra in 20% trifluoroethanol (TFE). In physiological buffer (PBS) containing lipid vesicles the MPG peptide transitions into a β-sheet structure with a broad minimum band in between 210 and 230 nm and a strong maximum band centered around 190 nm according to the CD spectrum. These peptide structures were retained when condensed with single- or double-stranded DNA, which resulted in effective cell permeability within human fibroblasts. Considering its efficient transfection capabilities of deoxyoligonucleotides, the MPG peptide has also been used to condense siRNA into stable nanoparticles that facilitated membrane translocation and efficient intracellular siRNA delivery in a variety of cell lines and *in vivo* (Crombez et al., 2007).

In an effort to improve cellular delivery and reduce immunogenic responses associated with nucleic acid delivery, MPG-8, was designed and developed as a shorter variant of the parent MPG peptide (Crombez et al., 2009). The 21-residue amphipathic peptide: bAFLGWLGAWGTMGWSPKKKRK-Cya contains a modified a β-alanine residue at the *N*-terminus to provide peptide stability and a spacer for further functionalization with a cholesterol moiety. The peptide formed stable non-covalent ionic complexes with siRNA targeting cyclin B1, which assembled into nanoparticles of varying sizes and distributions according to DLS measurements. Cell uptake within the HeLa cervical cancer cell line produced downregulation of cyclin B1 at the protein and mRNA levels of expression to a more significant extent relative to siRNA delivery using the parent MPG peptide. Consequently, downregulation of cyclin dependent kinase 1 (Cdk1-cyclin B1) signaling resulted in cell cycle arrest, and inhibition of cancer cell proliferation in HeLa, MCF-7, PC3, SKBr3-HER2 cancer cell lines. *In vivo* injection of the MPG-8-siRNA formulation within PC-3 bearing mice resulted in a significant (75%) reduction in tumor growth as a direct result of cyclin B1 downregulation. Furthermore, cholesterol functionalization improved significantly the *in vivo* biodistribution of siRNA while potentiating its tumor reduction capabilities relative to the non-functionalized MPG-8-siRNA formulation. The chol-MPG-8-siRNA rapidly dispersed into several tissues with an extended duration of action, even after 24 h plasma exposure. No treatment conditions triggered release of pro-inflammatory cytokines and chemokines in cell cultures and *in vivo* validating the safety efficacy of the MPG-8-siRNA formulation.

### Poly(lysine)

The poly(lysine) peptides are a class of cell penetrating peptides that have shown the capabilities to penetrate mammalian and bacterial cell types. This class of hydrophilic, polycationic homopeptide has similar properties to the poly(arginine) peptides with subtle changes in ionic and secondary structure compositions as well as its ability to condense polyanionic genetic material such as DNA and RNA for cell delivery. More specifically, poly(lysine) in comparison to poly(arginine) peptides demonstrated strong anti-cooperative binding and a 2-fold increase in dissociation constant (Kd values) with lipid bilayer mixtures consisting of phosphatidylglycerol and phosphatidylcholine according to fluorescence binding and molecular dynamic simulation studies (Robison et al., 2016). These results revealed that the cationic Lys side chain amino group interacts less strongly than the cationic Arg side chain guanidium group in the presence of negatively charged phospholipids. Moreover, poly(lysine) peptides also exhibited higher electrostatic repulsion between neighboring positively charged side chain amino groups which diminished their propensity to bind and interact favorably with the phospholipids. The ionic structure composition of poly(lysine) peptides may thus diminish its propensity to bind and penetrate cell membranes. However, secondary structure studies of poly(lysine) by electronic and vibrational CD spectroscopy revealed helix-to-coil transitions, that were dependent on temperature, solvent, additives and sample concentrations (Drake et al., 1988; Xia and Li, 2011). The apparent helical conformations were characterized as either a left-handed α-helix or a polyproline type II helix which primarily governed the most dominant peptide secondary structure motifs. This structural pre-organization has important consequences on cell permeability, as stable helical peptides have been favored in membrane translocation studies leading to their applications as cell penetrating peptides (Yamashita et al., 2017). Due to their ability to condense and release genetic material (Mann et al., 2011), poly(lysine) peptides have been used to transfect plasmid DNA (Mann et al., 2011) and siRNA (Mo et al., 2012) into cells for biological activity. Despite their utility, poly(lysine) peptides are still limited by their inferior cell binding and uptake capabilities, especially when compared to poly(arginine) peptides.

In order to improve their cell uptake, backbone modified poly(lysine) peptides substituting L-Lys for D-Lys residues have been generated synthetically, by Fmoc-based SPPS and demonstrated stable structures that were amenable to cellular translocation for gene delivery applications. For example, poly(D-lysine) has been used in nanomaterials composed of functionalized nanodiamonds with the ability to bind and condense therapeutic oligonucleotides consisting of unmethylated cytosine-phosphate-guanosine (CpG) that are commonly used as immune modulators against a variety of diseases, including cancer immunotherapy (Zhang et al., 2016). The poly(D-lysine) coated nanodiamonds effectively condensed CpG sequences into clusters of about 338 nm and with positive zeta potentials of 40 eV according to DLS studies. The cyanine dye Cy3-labeled nanoparticles were subjected to cell-based studies by confocal fluorescence microscopy, which demonstrated effective cell uptake via endosomes which were eventually co-localized with lysosomes following cytosol trafficking. Interestingly, fluorescence signaling from the nanoparticles was detected following 72 h incubation, with much slower clearance when compared to the uncoated nanodiamonds. Thus, poly(D-lysine) served to extend the intracellular resident time of the CpG sequences, thereby potentially increasing their duration of action. ELISA revealed the immunostimulatory activities of the functionalized nanodiamonds by the detection of tumor necrosis factor alpha (TNF-α), and interleukin-6 (IL-6), which were secreted to a greater extent and over an extended time period (72 h) when compared to the control CpG oligonucleotide alone. The *in vivo* immunostimulatory responses of the nanoparticles were also validated by the observed secretion of IL-6 and IL-12, even after 48 h treatment administration. The immunostimulatory effects led to significant tumor regression in B16-F0 melanoma (73%) and 4T1 breast (28%) tumor-bearing mice xenograft models. Comparatively, the CpG oligonucleotide treatment alone did not exhibit tumor reduction, presumably due to their poor metabolic stability and tumor uptake capabilities (Hanagata, 2012). Thus, poly(D-lysine) coated nanodiamonds function as efficient carriers of CpG oligonucleotides in cells and *in vivo*. Furthermore, no mice toxicity was observed despite nanoparticle biodistribution detected in the spleen, kidney, and lung validating its safe, effective use as an immunotherapeutic agent.

The hyperbranch or dendritic peptides encompass another related class of modified poly(lysine) motifs. They are built from either Fmoc or Boc-SPPS, respectively, using Fmoc-Lys(Fmoc)-OH and HATU/DIPEA or Boc-Lys(Boc)-OH and HBTU/HOBt/DIEA in DMF as the requisite coupling conditions for the divergent growth of poly(lysine) dendrons from each lysine branch point position (Al-Jamal et al., 2006; Janiszewska et al., 2016). Sample analyses and purifications were conducted with RP HPLC, while MALDI MS and NMR was used to confirm structure identities. Interestingly, the so-called 6th generation poly(lysine) dendrimer (Gly–Lys_63_(NH_2_)_64_) exhibited intrinsic fluorescence with excitation and emission wavelengths detected at 453 and 514 nm, respectively, according to spectrofluorimetry (Al-Jamal et al., 2006). Alternatively, linear, commercially available poly(lysine) hydrobromide (MW: 9600-121,000 Da) did not show any detectable fluorescence. The intrinsic fluorescence of poly(lysine) dendrimer was used to monitor cell uptake within Caco-2 cells. A time-dependent study (15 min−1 h) revealed membrane binding, translocation and rapid dispersion to all regions within the cell. Moreover, the poly(lysine) dendrimers were found to be non-toxic according to the MTT assay, validating their utility as safe, delivery agents.

Poly(lysine) dendigrafts have been effectively used to deliver siRNA and plasmid DNA *in vitro* and *in vivo* (Janiszewska et al., 2016). More specifically, they have been used to efficiently condense and shield genetic material from degradation. They have also facilitated delivery within the difficult to transfect glioma cells, which required peptide (EPRNEEK) conjugation for penetration of the blood-brain-barrier by laminin receptor-mediated endocytosis (Liu et al., 2014). This selective cell uptake property enabled *in vivo* biodistribution which revealed increased sample uptake in the brain, especially at the localized glioma site. The functional activity of plasmid DNA was revealed by the chemiluminescent reporter luciferase gene expression; while siRNA targeting the p42-mitogen activated protein kinase (MAPK) mRNA produced 35–40% knockdown; neither of which effected cell toxicity (Liu et al., 2014; Janiszewska et al., 2016).

## Selected Examples of Polyamide Backbone Modified Cell Targeting Peptides

### RGD

The RGD (Arg-Gly-Asp) peptide initially discovered in the early 1970s as a minimal binding motif for fibronectin (Ruoslahti and Pierschbacher, 1987), has since then been characterized as a binding ligand for naturally occurring α_v_β_3_ receptors. These also include integrin, fibronectin, vitronectin, plasminogen, thrombospondin, prothrombin, MMP-2, laminin, osteopontin, among other types of cell adhesion proteins (Danhier et al., 2012). The α_v_β_3_ integrin receptors are among the most studied class of cell adhesion proteins which modulate the interactions between the extracellular matrix and the cytoskeleton in many cell types. In cancer, the α_v_β_3_ integrin receptors are overexpressed and signal tumor proliferation, migration, and cellular adhesion rendering them resistant to many intervention strategies (Hosotani et al., 2002; Furger et al., 2003; Takayama et al., 2005; Vellon et al., 2007; Sheldrake and Patterson, 2009). Thus, the RGD peptide has been classified as a clinically relevant ligand for cell-targeting applications in the detection and treatment of $\alpha_{\text{v}}\beta_{3}^{+}$ cancers.

Despite its promising utility, the RGD peptide exhibits modest binding affinity and poor selectivity for the α_v_β_3_ integrin receptors, thereby reducing its clinical potential. Attempts to improve its receptor binding affinity and specificity were initially based on structure-activity relationship studies using NMR, molecular docking and X-ray crystallography of RGD analogs and their binding interactions at with the α_v_β_3_ integrin receptor (Auzzas et al., 2010). The cyclic RGD peptide analogs (cRGD) were among the very first modifications explored to improve α_v_β_3_ integrin receptor binding affinity and specificity. A lead (cRGDfV) identified from a spatial screening on a stereoisomeric library of RGD-containing peptides revealed a β type II'/γ conformation with Gly in the central position of the γ-turn and D-Phe at the i+1 position of the β type II'-turn (Kessler, 1997). This preorganized conformation positioned the side chains of Asp and Arg at optimal distances (~7 Å) for favorable receptor binding and inhibition. Since then, many cRGD analogs have been developed for cancer-based detection and treatment strategies. For example, the ^111^In- and ^99m^Tc-radiolabeled dimeric RGD peptides functionalized with 1,4,7,10-tetraazacyclododecane-1,4,7,10-tetraacetic acid (DOTA) and hydrazinonicotinamide (HYNIC) chelators (^111^In-DOTA-E-[c(RGDfK)]_2_) and ^99m^Tc-HYNIC-E-[c(RGDfK)]_2_) facilitated targeted tumor detection within NIH:OVCAR-3 xenograft mice models. Accumulation at the localized tumor site resulted in significant regression in tumor growth which was found to be α_v_β_3_ integrin-dependent when compared to the negative controls (Janssen et al., 2002). Similarly, a near infrared (NIR) multimeric cRGD peptide analog was designed and developed to enhance, visualize and quantify RGD molecular recognition of the α_v_β_3_ integrin receptor (Ye et al., 2006). In this application, the fluorescent NIR cyapte label was introduced onto multimeric (1–4 cRGD units) peptide scaffolds using Fmoc-SPPS with HOBt/HBTU as the preferred coupling reagents for peptide synthesis. The multimeric peptides were isolated by RP HPLC, characterized by ESI MS and subjected to competitive receptor binding studies to determine binding affinity (IC_50_ values) measurements. The multimeric cypate-labeled cRGD peptides exhibited sub-micromolar binding affinities which translated into cell-based and whole-body visualization of the peptides within A549 adenocarcinoma cells and xenograft mice models. Such was also found to be the case with FITC-labeled cRGD analogs, that possessed PEG and galactose groups for bioavailability and cell uptake in cancer cell lines and in tumor bearing mice models (Zheng et al., 2014). Interestingly, the FITC-labeled cRGD analogs improved αvβ3/αvβ5 receptor binding specificities when tested in a displacement assay with ^125^I-radiolabeled echistatin bound to U87MG glioma cells. The improved receptor binding specificities also resulted in enhanced cell uptake within colon, pancreatic, lung, squamous, gastric, and esophageal cancer which was also readily detected *in vivo*. In a newly developed cancer-targeted treatment strategy, the cyclic peptide iRGD (CRGDKGPDC) is cleaved by the neurophilin-1, a cell surface protease, which facilitates cell binding and penetration of the RGDK sequence (Sugahara et al., 2009). However, the short half-life and rapid renal clearance limits its clinical applications. In an effort to prolong its half-life and enhance its bio-activity, the iRGD peptide was formulated with N-(2-hydroxypropyl)methacrylamide (HMPA) as the biocompatible carrier, Dox as the anti-cancer drug for prostate cancer treatment and an MMP-2-degradable peptide linker (PLGLAG) which connected the iRGD peptide to the HMPA copolymer. The iRGD-based formulation exhibited good cell uptake, cell cycle arrest and cytotoxicity in DU-145 prostate cancer cell monolayers and within 3D multi-cellular tumor spheroids, underscoring its therapeutic utility in difficult to treat solid tumors (Peng and Kopeček, 2015). Recombinant cRGD has also been expressed on the surface of major coat proteins of filamentous phage, which in turn promoted integrin-dependent uptake in HeLa cells for potential applications in cancer diagnosis or therapy (Choi et al., 2014). cRGD has also been used to promote cell-dependent uptake of siRNA. In a recent application, multimeric cRGD conjugated to chemically modified luciferase siRNA was used to downregulate the reporter gene expression in $\alpha_{\text{v}}\beta_{3}^{+}$ M21^+^ human melanoma cells. Chemical conjugation of the multimeric cRGD analogs with Luc-siRNA was based on the Michael addition of a peptidic Cys thiol residue and the maleimide-functionalized siRNA. Sample analysis by LC-MS revealed degradation products at elevated temperatures (90°C) used for the siRNA annealing process. The proposed reaction mechanism was based on an intramolecular amine-assisted retro-Michael reaction that was avoided in sterile water. Cell uptake and subcellular localization of the AlexaFluor 488 conjugated cRGD-Luc siRNA samples revealed calveolae-depdendent endocytosis and endosomal uptake into the cytosol as the typical internalization mechanism of $\alpha_{\text{v}}\beta_{3}^{+}$ cells (Caswell and Norman, 2006). Furthermore, sample treatment did not result in any cell line toxicity, validating that the safe utility of cRGD as a α_v_β_3_ integrin-dependent transfection reagent for siRNA (Alam et al., 2011). Toward this effect, the bivalent cRGD conjugated to a methoxy-modified PIK3CB siRNA resulted in suppression of the phosphoinositide 3-kinases-protein kinase B-mammalian target of rapamycin (PI3K-AKT-mTOR)-signaling in glioblastoma, thereby inhibiting cell cycle progression and migration while enhancing tumor apoptosis *in vitro* and *in vivo* (Cen et al., 2018). Thus, cRGD analogs have served to effectively improve cancer cell line targeting and treatment compared to their linear counterparts.

The incorporation of rigid, privileged structural modifications has also been incorporated within the polyamide backbone of the RGD peptide to improve α_v_β_3_ receptor binding affinity and specificity. For example, the N-Me Val analog of cRGDfV, c(RGDf(NMe)V), also known as EMD121974 (Cilengitide) displayed potent and selective inhibition of the α_v_β_3_ integrin receptor, in part due to its preorganized 3D conformation observed in solution (Dechantsreiter et al., 1999). The peptide was found to adopt three turn geometries, two inverse γ-turns with Arg and Asp at i+1 position and a γ-turn with Gly at the i+1 position. The turn geometries resulted in a larger molecular spatial distance in between the Arg and Asp side chains, which in turn favored key binding interactions, including stable salt bridges in between the guanidium group of the Arg residue in c(RGDf(NMe)V) and Asp 150, Asp 218 residues found in the α subunit of the α_v_β_3_ integrin receptor (Marinelli et al., 2003). The compound's potent inhibitory activity toward the α_v_β_3_ integrin receptor facilitated its translation to cancer cell line testing, where it exhibited the most potent activity toward certain brain cancer types where it was found to be most effective as a single or in combination treatment regimens (Reardon et al., 2008). In spite of it success in advanced clinical trials, there is still little evidence of the compound's efficacy in other cancer types and its off-target binding to the α_v_β_5_ integrin receptor presents toxicity concerns. Since the discovery of Cilengitide, the search for new RGD-based privileged turn-inducing structures with improved pharmacological properties has resulted in the identification of several peptidomimetics. These include: azabicycloalkane and spiroheterocyclic systems (Haubner et al., 1996), macrocycles with quaternary stereogenic centers (Arosio et al., 2008), amino pyrrolidinone, and diketopiperazine motifs (Haubner et al., 1996; da Ressurreição et al., 2009), as well as morpholino (Cini et al., 2009) and carbohydrate (Lohof et al., 2000) containing RGD macrocycles, among many others (Auzzas et al., 2010). Thus, numerous polyamide backbone modifications have been designed and developed to improve the bio-activity and selectivity of RGD peptides for their corresponding integrin receptors.

### Pep42

The selection of Pep42, a disulfide-linked 13 amino acid containing peptide, CTVALPGGYVRVC, was derived from the whole cell panning of melanoma cancer cells which led to the phage display selection of the lead cell targeting peptide (Kim et al., 2006). The identified peptide clone, Pep42, displayed the capabilities to bind discriminately to metastatic melanoma cell lines, Me6652/4 but not to the control, non-metastatic melanoma variant, Me6652/56. Mut42, a derivative of Pep42 with substitution of Val (Garcia et al., 2018) for Lys enabled conjugation of the fluorescent FITC probe for studying cell uptake. The peptide was found to be cell permeable, with cytosol localization according to fluorescence microscopy and flow cytometry. Furthermore, in the presence of inhibitors of endocytosis, Mut42, was shown to exhibit energy-dependent receptor-mediated endocytosis within the metastatic Me6652/4 cells, but not within the control, non-metastatic melanoma variant, Me6652/56. Photoaffinity labeling of Mut42 with L-*p*-benzoylphenylalanine (Bpa), at the Lys-12 position with a biotin spacer produced the Mut42 derivative (Mut42-Bpa-biotin) that was susceptible to UV activation (λ_max_: 320 nm) for binding and isolation of the covalent peptide-receptor complex. Isolation of the cell lysates following peptide treatment and analysis by SDS PAGE and western blot revealed two bands, with isolated proteins of 80 and 130 kDa. Mass spectrometry analysis identified the 80 kDa band as GRP78, whereas, no significant signal was detected for the 130 kDa band. In order to confirm the GRP78-dependent binding of Pep42, quantum dots (QD) were conjugated to Mut42 and the nanoparticle formulation was added to the metastatic Me6652/4 cells, and the control, non-metastatic melanoma variant, Me6652/56. Cell uptake and perinuclear localization of the Mut42-QD was observed by confocal laser scanning microscopy to occur preferentially within the metastatic Me6652/4 melanoma cells but not within the control Me6652/56 cells and other non-cancer cell lines such as a human dermal fibroblast cell line. Cell uptake within the metastatic Me6652/4 melanoma cells was abrogated in the presence of the anti-GRP78 primary antibody, validating that Pep42 binding and cell uptake occurs in a GRP78-dependent manner. The binding and cell uptake mechanisms of Pep42 were further investigated in cell surface GRP78 expressing cancer cells such as lung adenocarcinoma (A549), osteosarcoma (SJSA-1), and the human hepatoblastoma (HepG2) cell line along with the GRP78^−^, non-tumorigenic fibroblast cell lines as controls. FITC-Pep42 bound selectively to the surface GRP78^+^ cancer cells and not to the surface GRP78^−^ controls, validating its GRP78-dependent cell binding activity. Cell uptake was also abolished in these cancer cell lines with pre-incubation of the anti-GRP78 primary antibody or with GRP78 silencing siRNA. Additional mechanistic studies into the cell uptake mechanism of Pep42 revealed that QD-Pep42 internalized within the A549 cells by a clathrin-mediated endocytosis mechanism which was not caveolae-dependent, lending further support to the GRP78-dependent mechanism for cell uptake. Treatment of the melanoma cancer cell lines with a Taxol-Mut42 conjugate resulted in cell-line dependent apoptosis which was contingent on cell surface GRP78 expression. The same cytotoxic effect on GRP78^+^ cancer cell lines was also found when Pep42 was conjugated with the pro-apoptotic D-(KLAKLAK)_2_ sequence or the photosensitizer, hematoporphyrin, triggering light-dependent phototoxicity. Within the Me6652/4 melanoma tumor xenograft mice model, QD-Pep42 was shown to accumulate preferentially at the localized tumor site, validating the tumor homing capabilities of Pep42 *in vivo* (Liu et al., 2007).

In order to improve the cancer-cell targeted delivery of toxic agents to cell surface GRP78 cancer cells, modified Pep42 derivatives have been designed and developed. For example, a pro-drug variant of Pep42, containing the valine-citrulline-*p*-aminobenzylalcohol (Val-Cit-PABOH) spacer which is rapidly cleaved by cathepsin B, was used to conjugate Taxol or Dox for controlled drug delivery within SJSA-1 osteosarcoma cells. The Pep42-drug conjugates displayed enhanced cytotoxicity within the SJSA-1 cells when compared to drug treatment alone (Yoneda et al., 2008). In a related application, a series of poly(D-arginine) sequences were added to the Pep42 sequence in order to improve its cell uptake and toxicity in HepG2 liver cancer cells. The FITC-labeled Pep42-poly(D-arginine) peptides displayed greater cell uptake compared to Pep42 and with greater efficiency with increasing poly(arginine) sequence. However, the poly(arginine) derived Pep42 peptides did not exhibit any significant cytotoxicity within the HepG2 cells (Joseph et al., 2014).

### SP5-52

The peptide SP5-52 (SVSVGMKPSPRP) was selected from an *in vivo* phage display targeting the tumor neovasculature but not normal blood vessels in severe combined immunodeficiency mice bearing human tumors (Lee et al., 2007). Structure-activity relationship studies revealed a consensus motif, proline-serine-proline, (Pro-Ser-Pro) that was found to be critical for peptide binding to the tumor neovasculature. More specifically, the SP5-52 peptide bound selectively to the vascular endothelial growth factor (VEGF) of human lung cancer surgical specimens, which resulted in tumor homing into eight different types of human tumor xenograft models. A liposome formulation consisting of SP5-52 peptide-linked Dox enhanced the therapeutic efficacy of the drug, decreased angiogenesis, and reduced tumor progression in human lung and oral cancer–bearing xenograft mice. Thus, the SP5-52-VEGF ligand-receptor interaction provides a novel basis for targeted therapy of the tumor neovasculature for potentially treating solid tumors and some types of blood cancers.

The retro-inverso D-peptide (prpspkmgvsvs) (D-SP5) is an analog of the parental peptide, SP5-52, which was used as a targeting ligand for gastric adenocarcinoma (SGC7901) cells (Li et al., 2015). The D-SP5 was functionalized with a PEG spacer containing a reactive maleimide group for conjugation with polyethyleneimine (PEI) to generate the D-SP5-PEG-PEI formulation which effectively condensed plasmid DNA (pGL) according to DLS and gel retardation assays. The D-SP5-PEG-PEI-pGL nanoparticle formulation showed effective transfection within SGC7901 cells according to quantitative luciferase detection of the pGL plasmid vector. Furthermore, flow cytometry revealed greater apoptosis of SGC7901 cells following treatment with D-SP5-PEG-PEI/pTRAIL when compared to the control, non-peptidic mPEG-PEI/pTRAIL formulation. This was attributed to the expression of the tumor necrosis factor-related apoptosis inducing ligand (TRAIL) protein according to western blot analysis. *In vivo* pharmacodynamic studies within the gastric adenocarcinoma SGC7901 xenograft mice models validated enhanced tumor regression (40%) with the D-SP5-PEG-PEI/pTRAIL treatment compared to the control, non-peptidic mPEG-PEI/pTRAIL formulation. Thus, the retro-inverso D-SP5 peptide provides a novel backbone modification to the parent SP5-52 peptide which resulted in an improved formulation with PEG-PEI, facilitating efficient gene delivery for therapy in gastric adenocarcinoma cells.

## Conclusions and Future Outlook

Cell penetrating/targeting peptides have been applied in the delivery of various cargos to malignant cell types, such as cancer, due to their capabilities of binding to cell surface markers and penetrating the cell membrane for intracellular activity. As such, CPPs and CTPs are promising tools for delivery of diagnostic as well as therapeutic agents inside cancer cells. However, their poor biochemical and pharmacological properties limit their therapeutic potential. Modified CPPs and CTPs have been designed and developed in order to improve their pre-clinical utility. Polyamide backbone modifications are among the most common types and have served to enhance the biological and biochemical characteristics of CPPs and CTPs in cancer cell lines, tissues and within tumor bearing xenograft models. Although promising results have been reported in pre-clinical *in vitro* and *in vivo* studies, no CPP or CTP analog has received FDA approval for clinical use. Therefore, additional studies are required to address their shortcomings in order to usher into the clinic a new wave of cell penetrating and targeting peptides for cancer diagnosis and therapy. This includes the continued development of medicinal chemistry programs for the discovery of new and improved peptidomimetics with advanced properties that addresses their clinical requirements. These include peptide modifications that: (i) improves plasma half-life stability, (ii) mediate high affinity and selective binding to onco-receptor targets, (iii) facilitates efficient cell permeability and endosomal escape within the tumor, and (iv) prolonged duration of action at the localized tumor site vs. minimizing host toxicity effects. In addition to peptide modifications, new carriers, additives and conjugates have been recently reported and are among the most promising precision nanomedicines available in pre-clinical and clinical studies. These are most often related to the development of translational conjugation or formulation techniques. For example, cancer-targeting peptides have been modified with synthetic linkers or spacers to facilitate bio-orthogonal conjugation of detection probes and anti-cancer agents such as siRNA that have led to cancer-targeted gene therapy *in vitro* and *in vivo* (Vrettos et al., 2018). Similarly, formulation strategies have been used for making peptide-based nanoparticles for the encapsulation and release of fluorescent probes and biologics directly within tumors for theranostic applications (Yoo et al., 2019). Thus, cell penetrating and targeting peptides remain at the forefront of current and future applications in cancer diagnosis and therapy.

## Author Contributions

SS, NC, GA, and DS contributed equally to this work by writing and revising the manuscript.

### Conflict of Interest

The authors declare that the research was conducted in the absence of any commercial or financial relationships that could be construed as a potential conflict of interest. The handling editor declared a past supervisory role with one of the authors DS.
